# Polypeptide Fraction from *Arca subcrenata* Induces Apoptosis and G2/M Phase Arrest in HeLa Cells via ROS-Mediated MAPKs Pathways

**DOI:** 10.1155/2015/930249

**Published:** 2015-05-21

**Authors:** Xianjing Hu, Zhang Zhang, Ting Liu, Liyan Song, Jianhua Zhu, Zhongyi Guo, Jinghua Cai, Rongmin Yu

**Affiliations:** ^1^Biotechnological Institute of Chinese Materia Medica, College of Pharmacy, Jinan University, 601 Huangpu Avenue West, Guangzhou 510632, China; ^2^Department of Pharmacology, College of Pharmacy, Jinan University, 601 Huangpu Avenue West, Guangzhou 510632, China; ^3^School of Chemistry and Chemical Engineering, Guangdong Pharmaceutical University, 9-13 Changmingshui Avenue, Wuguishan District, Zhongshan 528458, China

## Abstract

*Arca subcrenata* is documented in the literature of marine Traditional Chinese Medicine. Polypeptide fraction from *A. subcrenata*, coded as P2, was demonstrated to possess significant antitumor activity in our previous study. However, the underlying mechanism remains undefined. The present study was carried out to investigate the underlying antitumor mechanism of P2 in human cervical cancer HeLa cells by MTT, FCM, LSCM, and western blot assays. The results revealed that P2 significantly induced apoptosis of HeLa cells in a concentration- and time-dependent manner. High level of ROS was provoked by P2, which was in turn responsible for induction of apoptosis through activation of intrinsic mitochondrial pathway and JNK1/2, p38 MAPK pathways, as well as inhibition of ERK1/2 pathway, as evidenced by the abrogation of P2's effect on HeLa cells preincubated with the ROS scavenger NAC. P2 also was observed to display significant effect on G2/M phase arrest by downregulating the expression of cyclin B1/cdc2 complex and upregulating the expression of p21. These findings demonstrate that P2 induces apoptosis and G2/M phase arrest in HeLa cells through ROS-mediated MAPKs pathways, suggesting that P2 would be worth investigating as a promising agent within the scope of marine drugs for treatment of cervical cancer.

## 1. Introduction

Cervical cancer, the second most common type of tumor in females surpassed only by breast cancer [1], becomes a major health problem worldwide despite advances in screening [2]. For early-stage cervical cancer, patients are primarily treated by surgery or a combination of radiotherapy/chemotherapy [1]. Chemotherapeutic drugs are limited in clinical use because of their serious adverse effects, particularly with increased doses and prolonged courses [3]. Therefore, newer chemotherapeutic agents with fewer side effects and better efficacy are urgently needed for the treatment of cervical cancer.

Marine organisms are known for their capability of producing large amount of bioactive materials, with properties such as antitumor, anti-inflammatory, and antioxidant activities mainly due to the marine environment being characterized with high salinity, high pressure, low temperature, low light intensity, and an oligotrophic condition. Cytarabine, a nucleoside firstly obtained from sponge in the deep sea, was approved by FDA in 1969 and is regarded as one of the main drugs used to treat hematologic malignancies [4, 5]. Recently, numbers of marine-original compounds have been found to exhibit significant antitumor activity such as antiproliferation of bryostatins on the murine lymphocytic leukemia P388 cells [6], anti-invasion of sungsanpin on A549 cells [7], and apoptosis-induction of mycalamide A on HeLa cells [8]. Polypeptide originating from marine sources is an important substance that possesses antitumor effect owing to its high efficiency with specific cellular targets [9]. It is reported that some polypeptides from marine organisms could induce apoptotic death of cancer cells by triggering multiple signaling pathways [10] and arresting cell cycle at G1, S, or G2 phases [11, 12].

ROS, a series of natural metabolites of intracellular oxygen, plays a predominant role in cellular process and involves multiple signaling pathways as a second messenger [13], such as MAPKs pathway [14], NF-*κ*B pathway [15], and PI3K/AKT pathway [16]. A moderate level of intracellular ROS has the function of promoting cell proliferation or differentiation, while high level of ROS may result in genomic instability and toxicity to cancer cells [17] by inducing breakage in both single- and double-stranded DNA [18]. ROS would influence the MAPKs signaling pathways involved in cell survival and death [14]. Activation of JNK1/2 and p38 MAPK pathways would promote the release of cytochrome c and lead to a subsequent activation of caspases cascades for orchestrating responses to series of cellular stress [9, 19]. The ERK1/2 pathway, playing a key role in proliferation of tumor cells, has been recognized as an attractive target for a novel chemotherapy [20]. Numbers of marine-originated peptides, such as aplidine, theopederin B, and bisebromoamide, were found to exhibit significant antitumor activity by targeting MAPKs pathways in tumor cells [20–22]. Evidence has demonstrated that ROS plays an important role in cell cycle progression [13]. Low level of ROS results in the promotion of cell cycle progression, while high level induces growth arrest [13]. Numbers of anticancer drugs have been reported to induce apoptosis and cause cell-cycle arrest at the G2/M phase in neoplastic cells via over production of ROS [17, 23, 24].

*Arca subcrenata* Lischke, a member from Arcidae family, is classified as a marine invertebrate species [25]. It is widely distributed in the sea around China and easily accessible due to its low market price. Wa leng zi, a kind of Chinese Traditional Medicine, is made from the shell of* A. subcrenata* [25]. The body of* A. subcrenata* can be adopted in the treatment of a number of diseases such as inflammation, tumor, anemia, and dyspepsia in China [26].* A. subcrenata* is mainly composed of polysaccharide complexes and proteins [25, 26]. Recently, glycosaminoglycan obtained from* A. subcrenata* was reported to possess the bioactivity of improving the immunological function such as promotion of the thymus/spleen index of normal mice as well as immunosuppressed mice* in vivo* tests [27]. Our previous study showed that proteins from* A. subcrenata* could inhibit the proliferation of several tumor cell lines* in vitro* [28] and the hydrolysates of* A. subcrenata* had antioxidant activity against DPPH radicals and hydrogen peroxide [29].

More recently, we had confirmed that P2, a polypeptide fraction from* A. subcrenata*, displayed significant antitumor activities in both* in vitro* and* in vivo* tests [30]. However, the underlying mechanism of its antitumor activity remained to be characterized. We investigated the antitumor mechanism of P2 in HeLa cells in our current research and the data of which showed that P2 significantly induced apoptosis and G2/M phase arrest by highly increasing the level of ROS, which was responsible for activating intrinsic apoptosis pathway and JNK1/2, p38 MAPK pathways, as well as inhibiting ERK1/2 pathway. P2 was also found to significantly downregulate the expression of cyclin B1/cdc2 complex and upregulate p21 expression. These findings revealed important insight into the underlying antitumor mechanism of P2 in human cervical cancer HeLa cells.

## 2. Materials and Methods

### 2.1. Chemical Reagents and Antibodies

3-(4,5-Dimethylthiazol-2-yl)-2,5-diphenyltetrazolium bromide (MTT), N-acetylcysteine (NAC), Z-Val-Ala-Asp(OMe)-fluoromethylketone (Z-VAD-FMK), and 2′,7′-dichlorodihydrofluorescein diacetate (DCFH-DA) were purchased from Sigma (St. Louis, MO, USA). RPMI-1640 medium and fetal bovine serum (FBS) were purchased from Gibco (Grand Island, NY, USA). Primary antibodies against cleaved caspases 3, 7, and 9, PARP, p-cdc2, p21, cyclin B1, ERK1/2, p-ERK1/2, p38, p-p38, JNK1/2, p-JNK1/2, and anti-rabbit or anti-mouse IgG horseradish peroxidase- (HRP-) linked secondary antibodies were purchased from Cell Signaling Technology (Boston, MA, USA). Primary antibodies against Bax, Bcl-2, and cytochrome c were purchased from Abcam (Cambridge, UK). Primary antibody against GAPDH was from Hangzhou Xianzhi Biotech. Co., Ltd. (Zhejiang, China). Enhanced chemiluminescence (ECL) detection kit for western blot was obtained from Nanjing KeyGEN Biotech. Co., Ltd. (Jiangsu, China).

### 2.2. Preparation of Polypeptide Fraction from* A. subcrenata*

The material of* A. subcrenata* was purchased from Huangsha seafood market, Guangzhou, China, and identified by Professor Rongmin Yu (Jinan University, Guangzhou, China). The visceral mass of* A. subcrenata* was washed with distilled water for 3 times and homogenized with phosphate-buffered saline solution (1 : 3, w/v). The supernatant was collected after centrifugation and to which ammonium sulfate was added to precipitate the protein components. The crude polypeptides were further purified by ion-exchange chromatography eluted with different concentrations of sodium chloride solution at 4°C and the effluent fractions were detected using a protein detector at 280 nm. P2 was one of the effluent fractions with the highest content of protein. The collected fraction of P2 was concentrated, dialyzed against distilled water at 4°C, and lyophilized.

### 2.3. Cell Line and Culture

Human cervical cancer HeLa cells were purchased from the American Type Culture Collection (ATCC, Manassas, VA) and cultured in RPMI-1640 medium containing 10% FBS, 100 *μ*g/mL streptomycin, and 100 U/mL penicillin in a cell incubator with 5% CO_2_ at 37°C.

### 2.4. Cell Viability Assay

MTT assay was used to detect the viability of HeLa cells according to the literature with some necessary variations [30]. The cells were seeded at a density of 4 × 10^4^ cells/mL and incubated with P2 at various concentrations for setting time. The cells were pretreated by inhibitors for 1 h before treatment. Thereafter, MTT solution (5 mg/mL) was added and incubated with cells for another 4 h. The 96-well plates were added with 200 *μ*L/well of DMSO to dissolve the MTT-formazan product and the data were spectrophotometrically estimated at 570 nm. The cell growth inhibitory rate was determined according to the absorption.

### 2.5. Apoptosis Analysis

Apoptosis measurement was carried out mainly according to the literature [1] with some necessary variations. HeLa cells were treated with P2 at indicated concentrations for 12, 24, and 48 h. Then they were harvested (including attached and detached cells), washed, and resuspended with PBS. Apoptotic cells were determined with a FITC-Annexin V/PI Apoptosis Detection Kit (KeyGEN, Nanjing, China) by FCM (Becton Dickinson, Franklin Lakes, NJ). The percentage of apoptotic cells (including early apoptosis and late apoptosis) was analyzed by WinMDI 2.9 software.

### 2.6. DAPI Staining Assay

Cells were seeded at a density of 4 × 10^4^ cells/mL and incubated with 0, 4, 12, and 36 *μ*g/mL of P2 for 48 h. After the treatment with P2, the attached cells were washed twice with PBS and fixed with 4% formaldehyde for 10 min. The fixing solution was then removed and the cells were washed twice with PBS before staining with DAPI solution (Beyotime, Nanjing, China). After staining for 15 min in the dark, cells were washed again and observed under LSCM (Carl Zeiss, Thornwood, NY). Ten fields were randomly selected to observe and images were taken [17].

### 2.7. Cell Cycle Analysis

Cell cycle distribution analysis was performed according to the literature [12] with some variations. Briefly, cells were seeded at a density of 4 × 10^5^ cells/mL onto 6-well plates and incubated for 48 h with different concentrations of P2. After being treated with P2, cells were harvested and washed twice with cold PBS. Then cells were fixed in 70% alcohol for 12 h at 4°C. The pellet was washed and resuspended again in PBS containing PI (50 mg/mL), Triton X-100 (0.1%, v/v), and RNase A (100 mg/mL). After incubation for 30 min, the cell cycle distribution in each treatment was detected using a flow cytometer. Data were analyzed by WinMDI 2.9 software.

### 2.8. Mitochondrial Membrane Potential (MMP) Measurement

MMP of HeLa cells in each treatment was measured by FCM using JC-1 staining. Briefly, cells (4 × 10^5^/well) were seeded in a 6-well plate for 24 h. After being treated with different concentrations of P2 (0, 4, 12, and 36 *μ*g/mL) for 24 h, cells were harvested and washed twice with PBS and then incubated with JC-1 at 37°C for 30 min in the dark. Loss of MMP in HeLa cells was detected by measuring a change in fluorescence intensity using a flow cytometer, with excitation at 484 nm and emission at 501 nm [3].

### 2.9. Measurement of Intracellular ROS

DCFH-DA, a fluorescent probe of ROS, was used to measure the generation of intracellular ROS in P2-treated HeLa cells. Cells were seeded in 6-well plates (4 × 10^5^/well) for 24 h. After incubation, cells were treated with different concentrations of P2 (0, 4, 12, and 36 *μ*g/mL) for setting time. Then, the cells in each treatment were detached using trypsin-EDTA and washed twice with PBS. Cells were incubated with 10 *μ*M of DCFH-DA for 30 min in the dark. ROS level in each treatment was measured using a flow cytometer, with excitation at 485 nm and emission at 525 nm [17].

### 2.10. Western Blot Assay

The procedure of western blot was mainly performed according to the literature [23]. HeLa cells (including attached and detached cells) of all treatments were harvested and the proteins were extracted using RIPA lysis buffer supplemented with 1 mM of phenylmethanesulfonyl fluoride (PMSF) for 30 min on ice. Protein extracts were obtained by centrifugation at 4°C and the protein concentration was detected using bicinchoninic acid (BCA) protein assay kit (Beyotime, Nanjing, China). Equal amounts (30 *μ*g) of protein of each sample were separated on SDS-PAGE and further transferred to a methanol preactivated-polyvinylidene difluoride (PVDF) membrane (Millipore, Bedford, MA, USA). The membranes were blocked with 5% nonfat dry milk in Tris-buffered saline containing 0.1% Tween-20 (TBST) for 90 min at room temperature and incubated with primary antibodies at 1 : 400 to 1000 dilutions with 5% bovine serum albumin (BSA) in TBST overnight at 4°C. The blots were washed 3 times with TBST and incubated with HRP-conjugated secondary antibodies (1 : 2000) for 1 h at room temperature. Antibody binding was detected using ECL detection kit.

### 2.11. Statistical Analysis

Data were analyzed with Student's *t*-test and *P* < 0.05 was considered statistically significant. All results are representation of at least three independent tests and data are expressed as mean ± SD.

## 3. Results

### 3.1. P2 Induced Apoptosis of HeLa Cells in a Concentration- and Time-Dependent Manner

**Figure 1:**
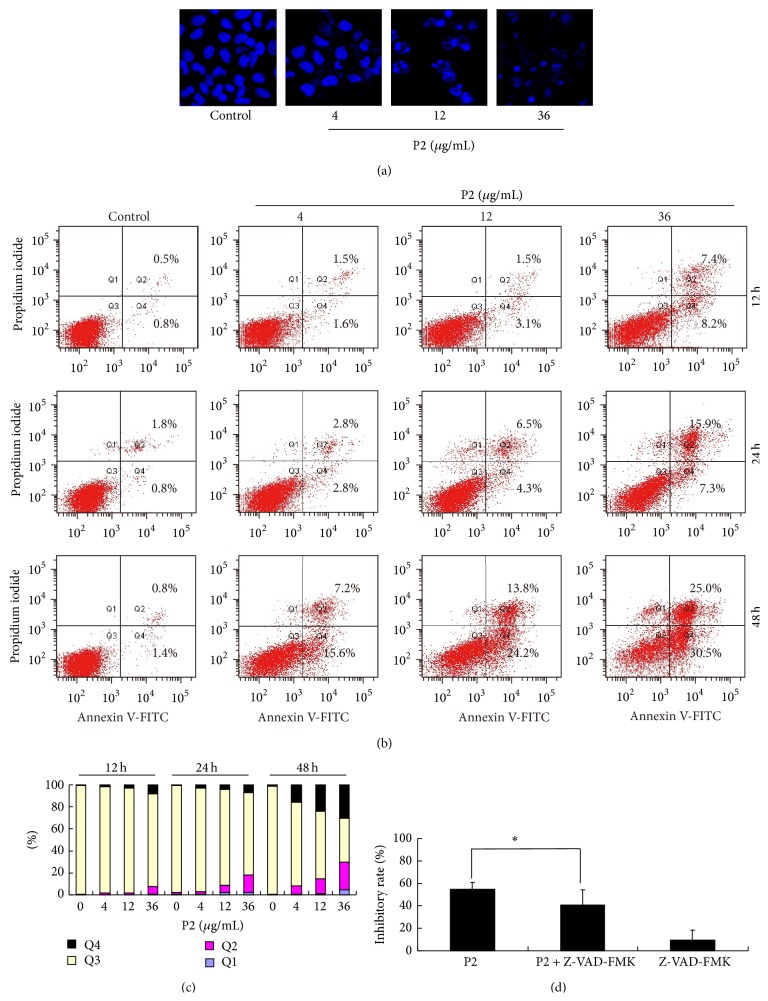
P2 effectively induced apoptosis of HeLa cells. (a) Morphological changes in HeLa cells after treatment with P2 (4, 12, and 36 *μ*g/mL) for 48 h. Cells were stained with DAPI and pictures were taken by LSCM. (b) Apoptosis analysis by FCM for annexin V/PI staining. HeLa cells were treated with P2 at different concentrations (0, 4, 12, and 36 *μ*g/mL) for 12, 24, and 48 h. (c) Histogram of apoptosis rates by P2 in the apoptosis-inducing test. (d) Antiproliferation of P2 on HeLa cells in a caspase-dependent manner. Cells were incubated with 20 *μ*M of Z-VAD-FMK for 1 h before treatment with P2 (12 *μ*g/mL) for 48 h. Cell inhibitory rate was assessed by MTT assay. Compared with P2 single treatment, ^*∗*^ *P* < 0.05 (*n* = 3).

Apoptosis, a means by which a number of chemotherapeutic agents kill cancer cells, is an important mechanism leading to cell death [31, 32]. To investigate the proapoptotic effect of P2, HeLa cells stained with DAPI were selected to analyze the morphological changes of apoptosis such as cell shrinkage, condensed and fragmented chromatin, and annexin V-FITC/PI double staining was used to quantify the percentage of apoptotic cells by FCM. As shown in Figure 1(a), condensed and fragmented chromatin and apoptotic bodies increased in P2-treated cells in a concentration-dependent manner. Apoptotic cells were further determined by calculating the percentage of early apoptosis (Annexin V^+^/PI^−^) and late apoptosis (Annexin V^+^/PI^+^) [33]. After being treated with 0, 4, 12, and 36 *μ*g/mL of P2 for 12, 24, and 48 h, both the percentages of early apoptotic cells and late apoptotic cells increased in a concentration- and time-dependent manner (Figures 1(b) and 1(c)). These results suggested that P2 could effectively induce apoptosis in HeLa cells.

It is reported that apoptosis was triggered by caspase-dependent or -independent way [14]. To clarify whether P2 exerted the antiproliferative effect on HeLa cells in a caspase-dependent manner, HeLa cells with pancaspase inhibitor Z-VAD-FMK were incubated for 1 h before being treated with 12 *μ*g/mL of P2. The result showed that Z-VAD-FMK significantly reduced the inhibitory effect of P2 on HeLa cells, indicating that P2's antiproliferative effect was caspase-dependent (Figure 1(d)).

### 3.2. P2 Triggered Intrinsic Apoptotic Pathway of HeLa Cells

**Figure 2:**
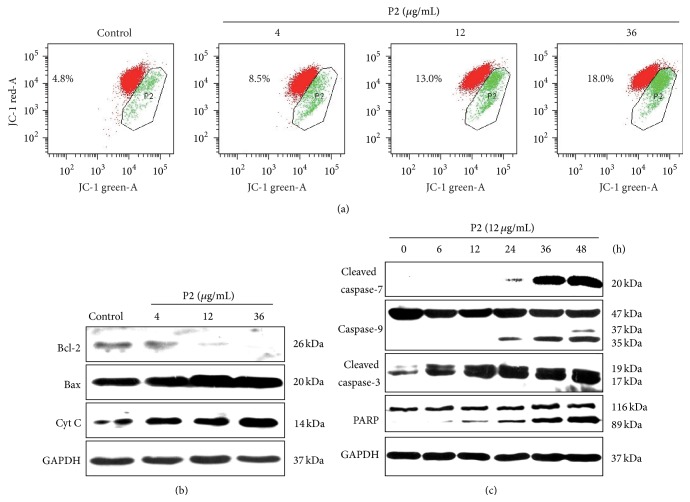
P2 triggered intrinsic apoptotic pathway of HeLa cells. (a) The mitochondrial membrane potential (Δ*φ*m) was analyzed by FCM. HeLa cells were treated with P2 (12 *μ*g/mL) for 24 h and Δ*φ*m was detected by a JC-1 detection kit. Red fluorescence stands for the mitochondria with an intact membrane potential and green fluorescence stands for the mitochondria with a disrupted membrane potential. (b, c) Western blot assay was adopted for detecting protein expression of the intrinsic apoptotic pathway in HeLa cells. The expression of Bcl-2, Bax, and cytochrome c was determined after HeLa cells were treated with 0, 4, 12, and 36 *μ*g/mL of P2, respectively, for 48 h (b). The expression of caspases 3, 7, and 9 and PARP was determined after HeLa cells were treated with 12 *μ*g/mL of P2 for 0, 6, 12, 24, 36, and 48 h, respectively (c). GAPDH was used as an internal control. Results shown are the representation of three independent experiments.

Apoptosis is commonly thought to be triggered through two major pathways, the intrinsic mitochondrial pathway and the extrinsic death receptor pathway [34]. To investigate the effect of P2 on the mitochondrial apoptotic pathway, the MMP of HeLa cells stained with the membrane potential sensor JC-1 was measured after being treated with P2. As shown in Figure 2(a), P2 obviously induced the reduction of the MMP in a concentration-dependent manner. The level of cytochrome c in the cytoplasm was further investigated by western blot assay. The result showed that cytochrome c in the cytoplasm increased in a concentration-dependent manner (Figure 2(b)), which corresponded to the decrease of MMP.

Bcl-2 family proteins consist of two types: proapoptotic proteins such as Bax, Bak, and Bcl-Xs and antiapoptotic proteins such as Bcl-2 and Bcl-XL [35]. Bcl-2/Bax ratio is recognized as a key factor in modulation of mitochondria-dependent apoptotic process due to its regulation on the release of cytochrome c from mitochondria to cytosol [36]. In the present study, the effect of P2 on expression of Bcl-2 and Bax was evaluated. As shown in Figure 2(b), the expression of Bcl-2 was downregulated while Bax was upregulated in a concentration-dependent manner in HeLa cells after treatment with P2 for 48 h, indicating that P2 induced apoptosis by shifting the Bax/Bcl-2 ratio in favor of apoptosis.

We further investigated the effect of P2 on expression of caspases 3, 7, and 9 and PARP, which are key factors in the mitochondrial apoptosis pathway [10]. As shown in Figure 2(c), the expression of full-length forms of caspase-9 and PARP slightly decreased while the cleaved forms of caspases 3, 7, and 9 and PARP significantly increased with the incubation time. The results demonstrated that P2 triggered the intrinsic mitochondrial apoptosis pathway of HeLa cells in a time-dependent manner.

### 3.3. P2 Effectively Induced G2/M Phase Arrest in HeLa Cells

**Figure 3:**
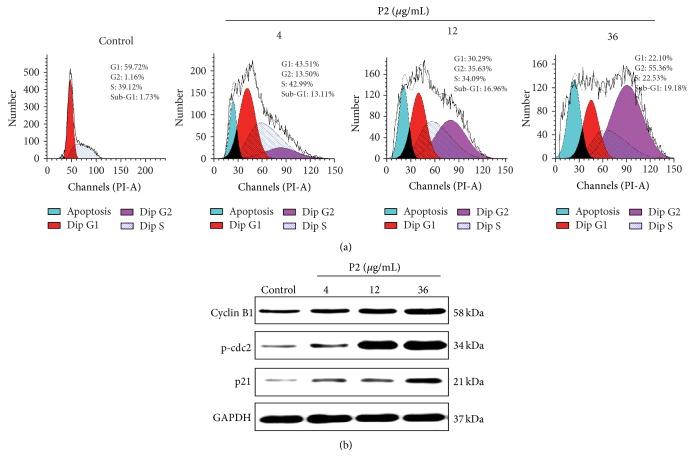
P2 effectively induced cycle arrest at G2/M phase in HeLa cells. (a) Effect of P2 on cell cycle distribution of HeLa cells. Cells were treated with 0, 4, 12, and 36 *μ*g/mL of P2 for 48 h and stained with PI. DNA contents in each treatment were then determined by FCM. Data showed a representative experiment in triplicate with similar results. (b) Effect of P2 on protein expression of cyclin B1, p-cdc2, and p21 in HeLa cells. Cells were treated with 0, 4, 12, and 36 *μ*g/mL of P2 for 48 h and the protein expression of cyclin B1, p-cdc2, and p21 was detected by western blot assay. GAPDH was used as an internal control. Results shown are the representation of three independent experiments.

Dysregulation of cell cycle is recognized to be a hallmark of tumor cells, and targeting the key proteins that control cell cycle would be a beneficial way in the antitumor strategies [23]. The effect of P2 on cell cycle progression in HeLa cells was further examined via flow cytometric analysis. As shown in Figure 3(a), the DNA contents of G2 phase in HeLa cells increased while those of G1/S phase significantly decreased in a concentration-dependent manner. The result also demonstrated that P2 caused visible accumulation of cells in sub-G1 peak, suggesting activation of apoptosis. It coincided with the result of the annexin V-FITC/PI double staining assay (Figure 1(b)). These results indicated that P2 inhibited the proliferation of HeLa cells via induction of apoptosis and G2/M phase arrest.

Cyclin B1 and cdc2 are involved in regulating G2 to M transition and the activation of cyclin B1/cdc2 complex triggers cells to enter mitosis from G2 phase [23]. Cdc2 is a kind of cyclin-dependent kinases (CDKs) and would be inactivated through phosphorylation by cdc25C [17]. In this study, the level of p-cdc2 was increased, suggesting the inactivation of cdc2 by P2 in HeLa cells (Figure 3(b)). Cells that have an increased level of cyclin B1 without possessing active cdc2 would fail to enter mitosis from G2 phase. p21, an important cyclin-dependent kinase inhibitor (CDKI), acting as a pivotal role in the induction of apoptosis and cell growth arrest, would be induced by both p53-dependent and p53-independent mechanisms [34, 37]. We further investigated the effect of P2 on the level of p21 in HeLa cells. Western blotting data showed that P2 upregulated the expression of p21 in a concentration-dependent manner, which would contribute to apoptosis induction and G2/M phase arrest (Figure 3(b)).

### 3.4. P2 Highly Enhanced the Intracellular Generation of ROS in HeLa Cells

**Figure 4:**
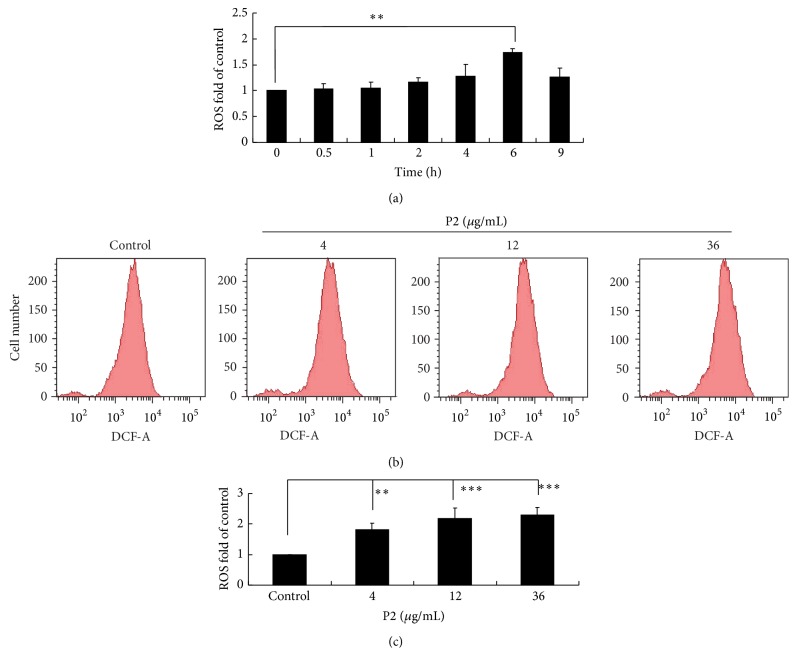
P2 highly enhanced the intracellular generation of ROS in HeLa cells. (a) Effect of P2 on ROS level in HeLa cells being treated for different time. Cells were treated with 12 *μ*g/mL of P2 for 0, 0.5, 1, 2, 4, 6, and 9 h, respectively, and detected for the fluorescence of DCFH-DA staining by FCM. Compared with the control, ^*∗∗*^ *P* < 0.01 (*n* = 3). (b, c) Effect of P2 at different concentrations on ROS level in HeLa cells. Cells were treated with 0, 4, 12, and 36 *μ*g/mL of P2 for 6 h and detected for the fluorescence of DCFH-DA staining. Compared with the control, ^*∗∗*^ *P* < 0.01, ^*∗∗∗*^ *P* < 0.001 (*n* = 3).

Overproduction of ROS is recognized to be an important mechanism of antiproliferation induced by chemotherapeutic agents in cervical cancer cells [38]. The intracellular generation of ROS in HeLa cells was subsequently analyzed by FCM. As shown in Figure 4(a), DCFH-DA-derived fluorescence of HeLa cells was showed to steadily increase, reaching a peak at 6 h after being treated with P2. In Figures 4(b) and 4(c), the ROS level significantly increased in a concentration-dependent manner, demonstrating that P2 enhanced the accumulation of ROS of HeLa cells in a concentration- and time-dependent manner.

### 3.5. P2 Activated JNK1/2, p38 MAPK Pathways and Inactivated ERK1/2 Pathway in HeLa Cells

**Figure 5:**
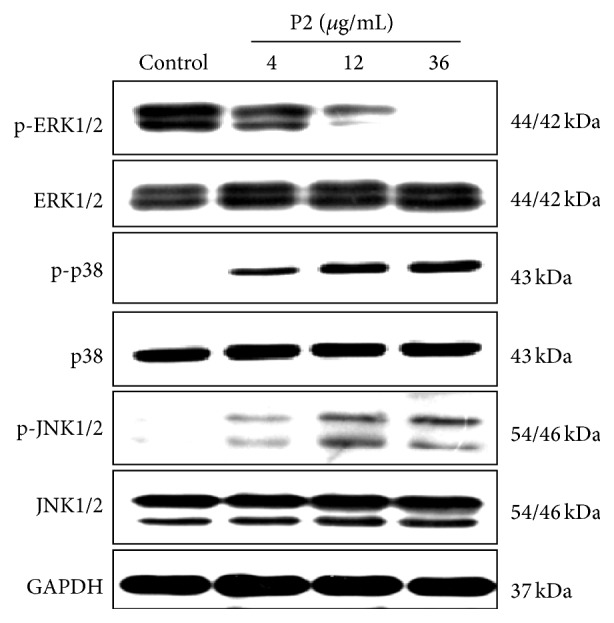
P2 activated JNK1/2, p38 MAPK pathways and inactivated ERK1/2 pathway in HeLa cells. Cells were treated with 0, 4, 12, and 36 *μ*g/mL of P2 for 48 h and the expression of ERK1/2, JNK1/2, p38 MAPK, p-ERK1/2, p-JNK1/2, and p-p38 MAPK was detected by western blot assay with GAPDH as an internal control. Results shown are the representation of three independent experiments.

MAPKs signaling pathways, including three major subfamily members JNK1/2, p38 MAPK, and ERK1/2 in mammals, would be activated by varieties of chemical and physical stresses [39]. The influence of P2 on MAPKs pathways in HeLa cells was then investigated at different concentrations. Western blot assay was used to detect the phosphorylated and nonphosphorylated levels of ERK1/2, JNK1/2, and p38 MAPK. There was no observable difference for P2 on the expression of ERK1/2, JNK1/2, and p38 as shown in Figure 5. On the other hand, the levels of p-p38 and p-JNK1/2 increased in a concentration-dependent manner with P2 treatment, while those of p-ERK1/2 decreased in a concentration-dependent manner.

### 3.6. ROS Played a Critical Role in P2-Inhibited Growth, -Induced Apoptosis, and -Modulated Effect on MAPKs Pathways of HeLa Cells

**Figure 6:**
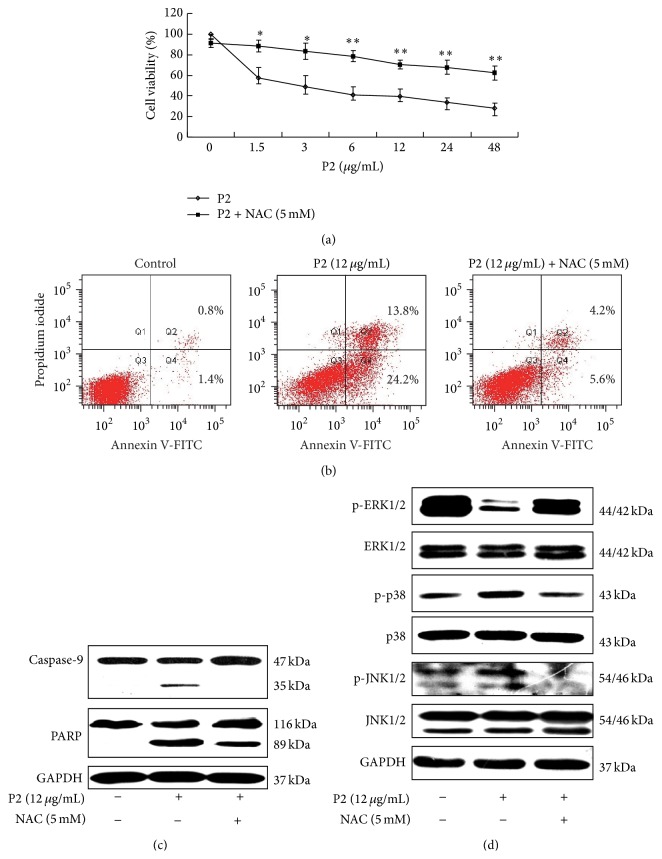
ROS played a critical role in P2-inhibited growth, -induced apoptosis, and -modulated effect on MAPKs pathways of HeLa cells. (a) NAC significantly attenuated the inhibitory effect of P2 on HeLa cells. Cells were treated with 5 mM of NAC for 1 h before treatment with 1.5, 3, 6, 12, 24, and 48 *μ*g/mL of P2 for 48 h. Cells inhibitory rate was assessed by MTT assay. Compared with P2 single treatment, ^*∗*^ *P* < 0.05, ^*∗∗*^ *P* < 0.01 (*n* = 3). (b) NAC abrogated the apoptosis-inducing effect of P2 on HeLa cells. Cells were treated with 5 mM of NAC for 1 h before treatment with 12 *μ*g/mL of P2 for 48 h. The rate of apoptosis was detected by FCM. (c) NAC markedly reversed the expression of apoptosis-related proteins including caspase-9 and PARP. Cells were treated with 5 mM of NAC for 1 h before treatment with 12 *μ*g/mL of P2 for 48 h and the expression of caspase-9 and PARP was determined by western blot assay with GAPDH as an internal control. Results shown are the representation of three independent experiments. (d) NAC attenuated the modulated effect of P2 on MAPKs pathways in HeLa cells. Cells were treated with 5 mM of NAC for 1 h before treatment with 12 *μ*g/mL of P2 for 48 h. The expression of ERK1/2, JNK1/2, p38 MAPK, p-ERK1/2, p-JNK1/2, and p-p38 MAPK was detected by western blot assay with GAPDH as an internal control. Results shown are the representation of three independent experiments.

ROS was reported to play a crucial role in some incidences of apoptosis [40]. NAC, an effective ROS scavenger, was used to pretreat the cells for 1 h for determining whether ROS played a pivotal role in the P2-inhibited growth and -induced apoptosis of HeLa cells. The result showed that NAC significantly attenuated the inhibitory effect of P2 on HeLa cells in a concentration-dependent manner (Figure 6(a)). Furthermore, we found that NAC abrogated the apoptosis-inducing effect of P2 on HeLa cells and markedly reversed the expression of apoptosis-related proteins such as caspase-9 and PARP (Figures 6(b) and 6(c)).

In order to uncover the relationship between ROS and MAPKs pathways, HeLa cells were pretreated with NAC prior to treatment with P2, and the protein expression levels of MAPKs were measured by western blot. As shown in Figure 6(d), the effect of P2 on the levels of phosphorylated MAPKs was markedly attenuated by NAC but those of nonphosphorylated MAPKs were not affected, suggesting that P2 could significantly increase the ROS level and regulate the MAPKs pathways including activation of JNK1/2, p38 MAPK and inactivation of ERK1/2 pathways.

## 4. Discussion

*A. subcrenata* has been used as a traditional medicine in China for thousands of years. In our previous study, P2, a peptide fraction obtained from* A. subcrenata*, was shown to have strong antitumor activities, with IC_50_ values of 11.43 *μ*g/mL against HeLa cells and 13.00 *μ*g/mL against HT-29 cells, as well as a tumor-growth-inhibitory rate of 60% in S-180 tumor-bearing mice at a dosage of 63 mg/kg [30]. In the current study, for the first time we investigated and elucidated the molecular mechanism of P2's antitumor effect on HeLa cells.

Apoptosis is a fundamental cellular process regulating cell development or differentiation and plays a key role in regulating proliferation and survival in normal and neoplastic cells [41]. Cells undergoing apoptosis have a series of variations in cellular process including chromatin condensation, DNA fragmentation, cell shrinkage, nuclear condensation, and phosphatidylserine (PS) flipping from the inner to the outer leaflet of the plasma membrane [42]. In this study, the result of morphological observation in P2-treated HeLa cells showed an emblematic feature with numbers of apoptosis bodies. The result was further confirmed in the apoptosis-rate-detecting assay. The intrinsic apoptotic pathway is often activated by various cell stress stimuli such as chemotherapeutic agents and radiation, which leads to altered ratio of Bcl-2 family members, promotes the loss of MMP and the release of cytochrome c as well as apoptotic protease activating factor-1 (Apaf-1) from mitochondria, and subsequently activates caspase-9 [43, 44]. Activated caspase-9 sets off further activation of downstream effectors of caspases 3 and 7, resulting in apoptotic cell death as detected in our study.

Mitochondria are both sources and targets of ROS [45]. Excessive amounts of ROS have been shown to cause peroxidative damage to the cell membrane [46], leading to subsequent change in the membrane permeability of mitochondria [47]. The mitochondrial malfunction provoked by high level of ROS in cells contributes to cell death by inducing apoptosis [40]. In our investigation, P2 was found to markedly increase ROS level. As a scavenger of ROS, NAC significantly attenuated the growth-inhibiting effect of P2, abrogated the apoptosis-inducing effect of P2, and reversed the expression of apoptotic proteins including caspase-9 and PARP in HeLa cells. These results steadily coincided with the verdict as declared.

It has been reported that MAPKs pathways can be triggered by ROS [14]. The activation of JNK and p38 MAPK signaling pathways could induce cell apoptosis while ERK protect cells from apoptosis by regulating Bcl-2 family proteins [48]. In the current study, P2 was found to upregulate the level of p-p38 and p-JNK1/2 and downregulate the level of p-ERK1/2 in HeLa cells. NAC was subsequently found to blunt the effect of P2 on expression of phosphorylated MAPKs but not nonphosphorylated MAPKs. Briefly, P2 led to a significant increase in the level of ROS, which then modulated MAPKs pathways by activation of JNK1/2, p38 MAPK and suppression of ERK1/2 to trigger intrinsic mitochondrial apoptotic death in HeLa cells.

**Figure 7:**
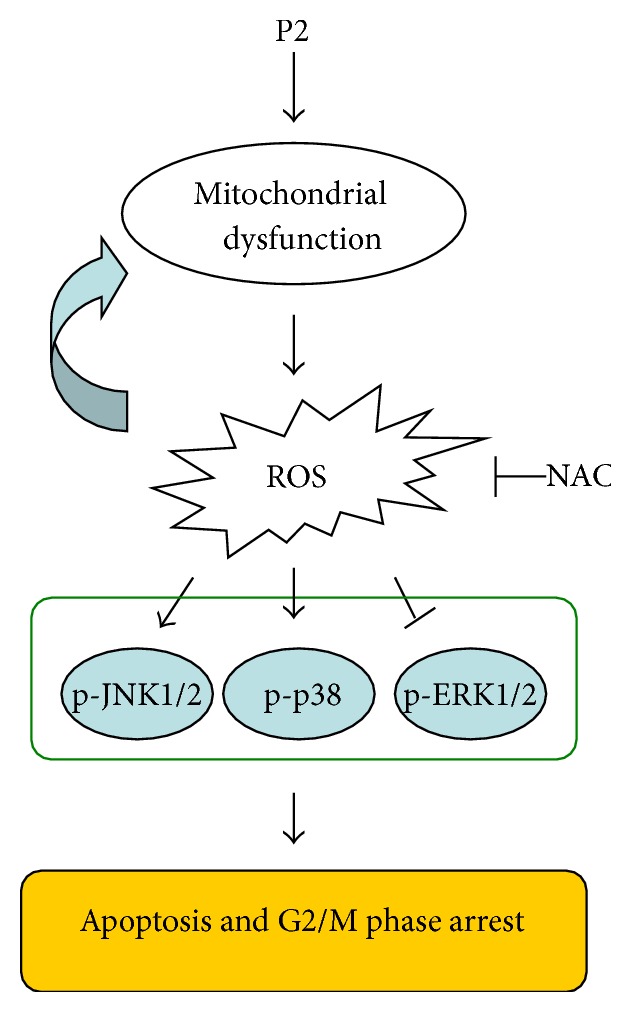
Proposed molecular mechanism of P2 included apoptosis induction and G2/M phase arrest via ROS-mediated MAPKs pathways in HeLa cells. → represents activation while ⊣ represents inactivation. The molecular mechanism of the antitumor activity of P2 was proposed according to the results obtained by MTT, FCM, LSCM, and western blot assays.

Cell growth and cell division are known to be controlled by several genetically defined cell-cycle checkpoints to ensure the correct progression of cell cycle through different stages [49]. Cell cycle checkpoints would also induce cell cycle arrest or activate DNA repair response when cells are simulated by varieties of stimuli [50]. The G2/M checkpoint was reported to play an important role for cell apoptosis caused by DNA damage [47, 51]. G2/M checkpoint could be a specific target for cancer therapy because of the preferential dependence on G2 over G1 checkpoint for DNA damage repair by cancer cells [49]. p21^waf1/cip1^, identified as a CDKI inducing cell cycle arrest, is a downstream target of p53 in the p53-dependent pathway and regulates DNA repair by interaction with proliferating cell nuclear antigen (PCNA) [13, 37]. In addition to arresting cell cycle, p21 also induces apoptosis via altering Bcl-2/Bax and activating caspases 9 and 8 [52]. ROS was reported to cause G2/M phase arrest mediated by MAPKs [13, 53] and promote p21 accumulation in p53-independent way in some cases [17, 54]. In our current study, P2 was demonstrated to highly increase the level of ROS and selectively induce G2/M phase arrest in HeLa cells, and the phenomenon was attributed to the decrease of cyclin B1/cdc2 complex, the key kinase provoking G2 phase into M phase. HeLa cells are genetically characteristic to be p53 protein deficient [17]. Our study revealed that p21 was probably activated in p53-independent pathway for inactivating cyclin B1/cdc2 complex in HeLa cells treated with P2. A proposed antitumor mechanism of P2 in HeLa cells was showed in Figure 7.

## 5. Conclusion

The current study, for the first time, demonstrates that P2 exerts antitumor effect via induction of apoptosis and cell cycle arrest at G2/M phase. The underlying mechanism is mainly related to ROS-mediated activation of JNK1/2 and p-38 MAPK pathways, inactivation of ERK1/2 pathway, and subsequent trigger of intrinsic mitochondrial apoptotic cell death in a caspase-dependent manner, including the activation of caspases 7, 9, and 3, and shifting the Bcl-2 family proteins. These results exhibit the exciting potential for development of antitumor agents from marine organisms through apoptosis induction and cell cycle arrest by ROS overproduction and targeting MAPKs pathways.

## Abbreviations

ROS: Reactive oxygen species
MAPKs: Mitogen-activated protein kinases
JNK1/2: c-Jun N-terminal kinase 1/2
ERK1/2: Extracellular signal-regulated kinase 1/2
FCM: Flow cytometry
LSCM: Laser scanning confocal microscope.
